# Interactions between cancer-associated fibroblasts and tumor cells promote MCL-1 dependency in estrogen receptor-positive breast cancers

**DOI:** 10.1038/s41388-018-0635-z

**Published:** 2019-01-10

**Authors:** K. Louault, T. L Bonneaud, C. Séveno, P. Gomez-Bougie, F. Nguyen, F. Gautier, N. Bourgeois, D. Loussouarn, O. Kerdraon, S. Barillé-Nion, P. Jézéquel, M. Campone, M. Amiot, P. P. Juin, F. Souazé

**Affiliations:** 1 CRCINA, Team 8, INSERM, Université d’Angers, Université de Nantes, Nantes, France; 2 SIRIC ILIAD, Angers, Nantes, France; 3 CRCINA, Team 10, INSERM, Université d’Angers, Université de Nantes, Nantes, France; 4 ONIRIS, Nantes Atlantic College of Veterinary Medicine Food Science and Engineering, Animal Cancers, Nantes, France; 5 ICO René Gauducheau, Saint Herblain, France; 6 Service d’Anatomie Pathologique, CHU Nantes, Nantes, France; 7 CNRS GDR3697 Micronit, Tours, France

**Keywords:** Apoptosis, Breast cancer

## Abstract

Selective inhibition of BCL-2 is expected to enhance therapeutic vulnerability in luminal estrogen receptor-positive breast cancers. We show here that the BCL-2 dependency of luminal tumor cells is nevertheless mitigated by breast cancer-associated fibroblasts (bCAFs) in a manner that defines MCL-1 as another critical therapeutic target. bCAFs favor MCL-1 expression and apoptotic resistance in luminal cancer cells in a IL-6 dependent manner while their own, robust, survival also relies on MCL-1. Studies based on ex vivo cultures of human luminal breast cancer tissues further argue that the contribution of stroma-derived signals to MCL-1 expression shapes BCL-2 dependency. Thus, MCL-1 inhibitors are beneficial for targeted apoptosis of breast tumor ecosystems, even in a subtype where MCL-1 dependency is not intrinsically driven by oncogenic pathways.

## Introduction

Breast cancer is a heterogeneous disease that encompasses distinct molecular subtypes. The majority of mammary carcinomas express, in a significant portion of malignant cells, hormone receptors for estrogen (ER) and/or progesterone (PgR). These breast cancers include luminal A ones (LUM A), and luminal B ones (LUM B), less prevalent but significantly more aggressive and of poor prognosis. Patients are treated by endocrine therapy, with, in general, additional chemotherapy for LUM B. As therapy resistance remains a major obstacle to the successful treatment of these cancers new more efficiently cytotoxic approaches need to be identified.

Proteins of the BCL-2 family critically regulate the mitochondrial apoptotic pathway by engaging a network of intracellular interactions through which BCL-2 homologues (BCL-2, BCL-xL, and MCL-1) exert distinct, complementary anti-apoptotic activities [1]. These activities need to be overcome for therapy to be efficient. Reciprocally, direct inhibition of anti-apoptotic proteins may suffice to enhance cancer cell vulnerability and promote apoptosis [2]. Targeted mitochondrial apoptosis is now achievable by selective BH3 mimetic inhibitors. ABT-737 (a preclinical lead compound) and its orally available equivalent ABT-263 (Navitoclax) bind to and inhibit BCL-2 and BCL-xL but not MCL-1, while ABT-199 (Venetoclax) preferentially targets BCL-2. The latter compound presents the clinical advantage to avoid dose-dependent thrombocytopenia, a side-effect of the former ones related to the BCL-xL dependency of mature platelets. It was recently approved for the treatment of chronic lymphocytic leukemia and it is also promising in other hematological malignancies [3].

Several lines of evidence indicate that the BCL-2 inhibitory activity of the above compounds might be useful to enhance the vulnerability of luminal breast cancers. BCL-2 is a direct transcriptional target of ERα [4], and its mRNA and protein levels are predominantly expressed in ER-positive cancers [5, 6]. Moreover, in preclinical patient-derived xenograft (PDX) models of primary luminal B cancers, ABT-737 and ABT-199 were shown to have pro-apoptotic activity [7]. Further characterization of tumor features that modulate BCL-2 dependency is however required to clearly define the clinical utility of these BH3 mimetics. Their efficiency is indeed subject to multiple regulatory steps, as it relies on the establishment of a “mito-primed” state in target cells (dependent upon expression and activity of pro-apoptotic BCL-2 family members) and on the relative balance between expressions of the targeted anti-apoptotic proteins and the non-targeted ones (typically MCL-1 in the case of ABT-737 and ABT-199 [8]).

The question of the stromal influence on BCL-2 dependency is particularly apposite in breast cancer given the established impact of a hijacked stroma on this disease progression and therapeutic response. Cancer-associated fibroblasts (CAFs) make up the bulk of cancer stroma and they may represent up to 70% of the whole breast tumor volume. Specific CAF features might serve as biomarkers to refine clinical diagnosis, prognosis and therapeutic choice [9, 10] and expression of stromal genes is of poor prognosis [10]. Most relevantly here CAFs were shown to contribute to endocrine and chemotherapy resistance in a paracrine manner [11]. The spatial proximity to CAFs promotes, in particular, cancer cell resistance to doxorubicin and paclitaxel, two compounds frequently used in the chemotherapeutic regimen of breast cancers, including LUM B ones [12]. In this manuscript, we thus explored whether BCL-2 inhibition would bypass the protective effects of CAFs, how CAFs might mitigate BCL-2 dependency otherwise and whether additional vulnerabilities might ensue from the CAFs/luminal cancer cell interactions.

## Results

### bCAFs reduce BCL-2 dependency in luminal breast cancers by paracrinely favoring MCL-1 expression

**Fig. 1:**
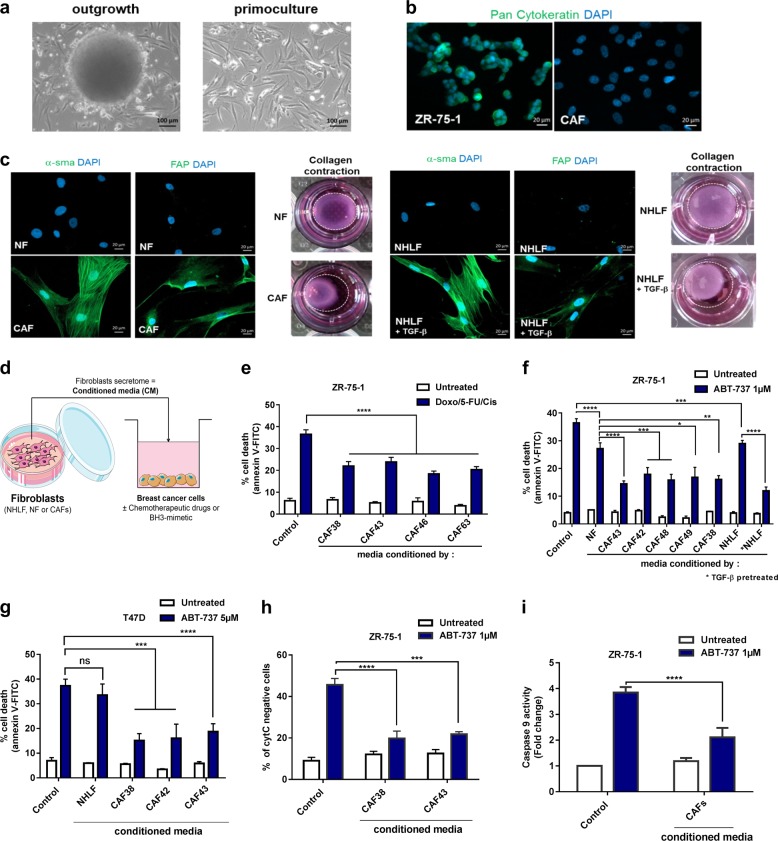
bCAFs reduce BCL-2 dependency in luminal breast cancers. **a** Outgrowth of CAFs from partially enzymatically digested tissue of human breast tumor resections (left panel) and the resulting in vitro primo-culture (right panel) showing a homogenous fibroblastic phenotype. **b** Immunofluorescence of Pan Cytokeratin (green) in bCAFs and ZR-75-1 cell line, nuclei were stained in blue (4′,6-diamidino-2-phenylindole, DAPI). **c** Immunofluorescence of α-smooth muscle actin (α-SMA) and Fibroblast activation protein (FAP) (green) in NF and CAF (left panel) and in NHLF+ /- TGF-β (right panel), nuclei were stained in blue (4′,6-diamidino-2-phenylindole, DAPI). Images of fibroblasts (NF, CAF, left) or (NHLF+ /- TGF-β, right) contraction of collagen gels after 3 h. **d**-**i** Protective effects of media conditioned by bCAFs on ZR-75-1 cells (**e**, **f**, **h**, **i**) or T-47D cells (**g**). The indicated tumor cells were treated for 48 h with (**e**) Doxorubicin (2.5 µM)/5-Fluorouracil (27.5 µM)/Cisplatin (55 µM) or (**f**, **g**, **h**, **i**) ABT-737 (1 µM or 5 µM, as indicated) in presence of non-conditioned media (Control) or media conditioned for 48 h by normal fibroblast (NF), normal human lung fibroblast (NHLF) (pre-treated or not by TGFβ, overnight), or cancer-associated fibroblasts (CAFs) as indicated. Percentage of positive Annexin-V-FITC apoptotic cells was measured by flow cytometry in **e**, **f**, **g**; percentage of cytochrome C negative cells was measured by flow cytometry in **h**; Caspase 9 activity was measured by caspase Glo assay and expressed as fold change relative to the control (untreated w/o CAFs conditioned media) in **i**. Data are means ± SEM from three independent experiments. *P*-value was determined by two-way ANOVA. ***P < 0.001, ****P < 0.0001, ns: not significant

**Fig. 2:**
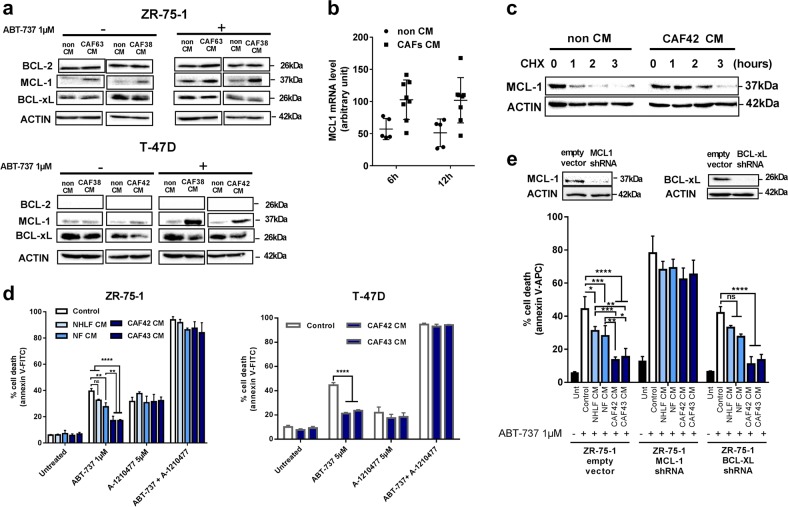
bCAFs exert their protective effects by paracrinely favoring MCL-1 expression. **a** Anti-apoptotic (BCL-2, MCL-1, BCL-xL) proteins expression levels in ZR-75-1 (top) or T-47D (bottom) cells, treated or not for 24 h with ABT-737 (1 µM) in presence of non-CM or CAFs-CM, were evaluated using western-blot analysis. **b** qRT-PCR of *MCL-1* mRNA in ZR-75-1 cells grown in presence of non-CM or different CAFs CM for six and 12 h. Mean and SEM of three independent experiments are represented as relative quantity of mRNA normalized to the mean of *RPLP0*, *B2M* and *GAPDH* relative expression. **c** MCL-1 proteins expression levels in ZR-75-1 cells grown in presence of non-CM or CAFs-CM for 24 h and treated by cycloheximide for the indicated time, were evaluated using western-blot analysis. **d** ZR-75-1 cells (left) or T-47D cells (right) were treated for 48 h with ABT-737 1 µM and/or A-1210477 5 µM in presence of non-CM or Fibroblasts-CM. **e** ZR-75-1 cells infected by empty vector, MCL-1 or BCL-X_L_ shRNA, were treated for 48 h with ABT-737 1 µM in presence of non-CM or fibroblasts-CM. Percentage of positive Annexin-V-FITC or –APC apoptotic cells was measured by flow cytometry (**d**, **e**). Data are means ± SEM from three independent experiments. *P*-value was determined by two-way ANOVA. *P < 0.05, **P < 0.01, ***P < 0.001, ****P < 0.0001, ns: not significant

To analyze bCAFs functionally, we isolated and characterized primary cultures of fibroblasts isolated from nine surgical resections of treatment naïve luminal breast carcinoma (Fig. 1a–c Supplementary Table 1, Supplementary fig. 1a, b and Supplementary information [13]). To investigate possible paracrine pro-survival activity, we prepared bCAFs conditioned media (CM) (Fig. 1d) and measured their effects on the apoptotic response of Erα positive breast cancer cell lines. In ZR-75-1 cells, bCAFs-CM promoted resistance against apoptosis induction by a 5-Fluorouracil/Doxorubicin/Cisplatin combination chemotherapeutic treatment (Fig. 1e). This was not bypassed by BCL-2 (plus BCL-xL) inhibition as bCAFs- CM also inhibited apoptosis induced by the BH-3 mimetic BCL2/BCL-xL inhibitor ABT-737 (Fig.1f). CM derived from normal human lung fibroblast (NHLF) or normal fibroblast (NF) have less protective effects, in contrast to CM derived from TGFβ-pretreated NHLF (an experimental paradigm that roughly approximates the conversion of fibroblasts into CAFs [14], see also Fig. 1c and Fig. S1c). Similar results were obtained in the T47D luminal breast cancer cell line (Fig. 1g). Triggering of the intrinsic pathway of apoptosis by ABT-737, and its prevention by bCAFs-CM, were confirmed by cytochrome c release and CASPASE-9 activation assays (Fig. 1h, i). Notably, bCAFs-CM also protected cancer cells from apoptosis induced by ABT199 + /- fulvestrant (Fig. S1d, i) or ABT-199 + /- chemotherapy (Fig. S1e). Moreover, treatment of ZR-75-1 cells with the selective BCL-xL inhibitor WEHI-539 triggered no detectable apoptosis by itself (Fig. S1f), and BCL-xL downregulation in ZR-75-1 cells by a lentiviral-based shRNA approach neither abrogated ABT-737 induction of cell death in the absence of CM, nor the protective effects of bCAFs-derived CM (Fig. 2e). We cannot rule out, from the last experiment, that the enhanced pro-apoptotic effects of ABT-737 compared to that of ABT-199 are due to the inhibition of a third target (such as BCL-w) in addition to BCL-2 and superfluous BCL-xL. Nevertheless, our data strongly argue that: (i) BCL-2 plays a critical role in the survival of luminal breast cancer cells; (ii) CAFs produce soluble factors that erode the apoptotic consequences of inhibition of this critical protein, even if tumor cells receive endocrine therapy or chemotherapy. Of note, the protective effects of bCAFs-CM were not bypassed by addition of Herceptin to ZR-75-1 (that was claimed to express HER2 [15]) indicating that targeted inhibition of HER2 (when eligible) would also fail to override stroma-induced resistance (Fig. S1g–i).

To hint on the most clinically relevant mechanism(s) involved in the protective effects described above, we first explored the publicly available luminal breast cancer expression TCGA dataset and sought for co-variations between a short list of apoptosis-related genes and a stromal score (determined by the ESTIMATE algorithm and used as a gross surrogate of the presence of bCAFs, that are the major constituents of breast cancer stroma). As shown in Table 1, MCL-1 was the only anti-apoptotic gene whose mRNA expression positively correlated with the stromal score (Table 1, bold). Based on these premises, we measured MCL-1 expression changes in luminal cancer cells upon addition of bCAFs -CM. These CM-induced MCL-1 expression at both mRNA and protein level in ZR-75-1 cells and this induction was maintained when cancer cells were challenged by ABT-737 (Fig. 2a, b). Protein expression of BCL-2 and BCL-xL were, in contrast, left unchanged (Fig. 2a top). A similar effect on MCL-1 protein level was detected in T-47D cell lines (Fig. 2a, bottom). As MCL-1 protein has a short half-life, we also investigated whether bCAFs-CM impact on its turnover using cycloheximide (CHX), an inhibitor of protein biosynthesis. In CM exposed cells, MCL-1 levels remained stable during CHX treatment significantly longer that in control cells (Fig. 2c). Thus bCAFs favor both MCL-1 mRNA expression and protein stability in cancer cells. This most likely contributes to bCAFs induced resistance to BCL-2/BCL-xL inhibition, as the BH3 mimetic MCL-1 inhibitor A-1210477 [16] reversed the protective effects of bCAFs -CM against ABT-737 (Fig. 2d). Further supporting this notion, downregulating MCL-1 expression by shRNA in ZR-75.1 cells (in contrast to that of BCL-xL as reported above) rendered cells insensitive to the protective effects of bCAFs-CM (Fig. 2e). Altogether, these results show that factors secreted by CAFs led to MCL-1 induction and MCL-1 dependent resistance to the apoptotic effects of BCL-2 inhibition in the neighboring breast cancer cells.

**Table 1:** Correlations between stromal score and apoptosis-related gene mRNA expression from TCGA data portal

GENE	Pears
CASP3	0.10101
BCL2L11	−0.09442
BCL2	−0.16006
BID	0.19328
APAF1	−0.21429
CASP7	0.28268
**MCL1**	**0** **.30414**
PMAIP1	−0.06278
CASP10	0.54548
BAD	−0.10497
BAX	0.00835
CASP6	0.00365
CASP9	−0.0274
BCL2L1	−0.20332
BAK1	0.07807
CASP8	−0.06217

Pearson’s *r* was calculated using the Affymetrix® expression levels of apoptosis-related genes of 321 TCGA Breast tumor samples (Luminal A/B, Nature, 2012 and the corresponding stromal scores. The results shown here are based upon data generated by the TCGA Research Network: http://cancergenome.nih.gov.gate2.inist.fr/ [39]

### bCAFs produced IL-6 contributes to their protective effects

**Fig. 3:**
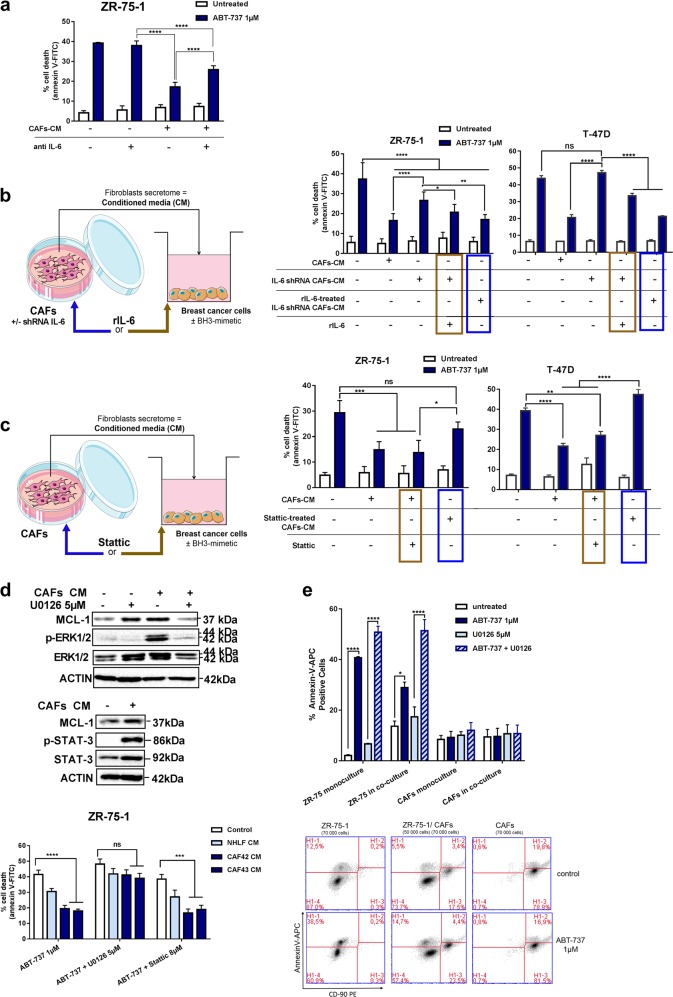
bCAFs produced IL-6 contributes to their protective effects. **a** ZR-75-1 cells were treated for 48 h with ABT-737 (1 µM) in presence of non-CM or CAFs-CM with or without IL-6 neutralizing antibody (anti IL-6). **b** ZR-75-1 or T-47D cells were treated for 48 h with ABT-737 (1 µM) in presence of non-CM, control (empty vector) or IL-6 shRNA CAFs-CM. For rescue, recombinant IL-6 (rIL-6; 500 pg/ml) was added on cancer cells within the CM (IL-6 shRNA + rIL-6) or previously during the media conditioning (IL-6 shRNA in presence of rIL-6) (as schematized in the bottom right panel). **c** ZR-75-1 or T-47D cells were treated for 48 h with ABT-737 1 µM in presence of non-CM, CAFs-CM (untreated), CAFs-CM co-treated with Stattic 8 µM (Stattic 8 µM) or CAFs-CM pre-incubated with Stattic 8 µM during CAFs media conditioning (Stattic pre-incubated). **d** Top: MCL-1, P-ERK, and ERK expression levels were evaluated by western-blot in ZR-75-1 cells treated for 24 h with ABT-737 (1 µM) alone or in combination with U0126 (5 µM) in presence of non-conditioned media or CAF-conditioned media. MCL-1, P-STAT-3 and STAT-3 expression levels were evaluated by western-blot in ZR-75-1 cells treated for 24 h with ABT-737 (1 µM) in presence of non-conditioned media or CAF-conditioned media. Bottom: ZR-75-1 cells were treated for 48 h with ABT-737 (1 µM) alone or in combination with U0126 (5 µM) or Stattic (8 µM) in presence of non-conditioned media or fibroblasts-conditioned media (NHLF, NF, CAFs). Percentage of positive Annexin-V-FITC cells was measured by flow cytometry. **e** In a co-culture model, ZR-75-1 cells (1/3) and CAFs (2/3) were treated with ABT-737 1 µM and U0126 5 µM alone or in combination, in DMEM 0.5% FBS for 48 h. CAFs were stained with anti CD-90 PE (bottom). Percentage of positive Annexin-V-APC apoptotic cells was measured by flow cytometry in each of the two cell populations (ZR-75 or CAF) (top). Data are means ± SEM from three independent experiments. *P*-value was determined by two-way ANOVA. *P < 0.05, **P < 0.01, ***P < 0.001, ****P < 0.0001, ns: not significant

We established that protective factor(s) produced by CAFs are thermo-labile and present in the soluble (and not extracellular vesicle) fraction (see Supplementary information and Fig. S2a, b, c). As a first step to sort out factors involved, we therefore searched for cytokines whose mRNA expression positively correlated with that of MCL-1 expression in the luminal breast cancer expression data set used above (Supplementary information). Out of the 16 cytokines identified (Supplementary Table 2), IL-6 attracted our attention as a candidate factor as we evaluated it to be highly secreted by bCAFs, and by NHLF after their “activation” by TGFβ (Fig. S3a). In contrast human luminal breast cancer cells produce no detectable IL-6 (Fig. S3a and [17]), and thus could benefit from IL-6 only paracrinely. Several lines of evidence incriminated this cytokine as one factor directly impacting on cancer cells resistance. Firstly, the addition of recombinant IL-6 (rIL-6) to cancer cells decreased their response to ABT-737 (Fig. S3b). Secondly, blocking IL-6 activity in bCAFs-CM using a specific antibody lead to a significant reversion of the CM protective effect (Fig. 3a). Thirdly, conditioned media from CAFs in which IL-6 was downregulated by shRNA (Fig. 3b left) (leading to a 75% decrease in soluble IL-6 concentrations in CAFs-CM as measured in ELISA, Fig. S3c) were less protective than control bCAFs-CM (Fig. 3b right).

We noted, however, that the reversing effects of anti-IL-6 inhibitory antibodies in CM were only partial, and that addition of rIL-6 to CM from IL-6 downregulated CAFs did not restore full protection (Fig. 3a, b). In sharp contrast, the rescue was complete when rIL-6 was added to IL-6 depleted CAFs during medium conditioning (Fig. 3b; rIL-6 -treated IL-6 shRNA CAFs-CM). Thus IL-6 also functions as an autocrine factor to promote the production by CAFs of factor(s) that add their effects on these of IL-6 on cancer cells. In full support to this, treatment of CAFs with the STAT3 inhibitor, Stattic, to interfere with IL-6 autocrine signaling during the conditioning of the media prevented their protective effects (Fig. 3c) without altering IL-6 production (Fig. S3d) and without impacting itself on cancer cells survival (see below).

IL-6 was shown to exert MCL-1 anti-apoptotic effects via the STAT-3 and/or MAP kinase signaling pathways [18] that regulate MCL-1 at the transcriptional and post-translational level respectively [9]. As shown in Fig. 3d (top) CAFs-CM-induced both phosphorylation of ERK and STAT-3 in cancer cells. ERK inhibition by addition of U0126 to cancer cells counteracted bCAFs-CM protection against induction of cell death by ABT-737 whereas STAT-3 inhibition using Stattic did not (Fig. 3d, bottom). Under these conditions, U0126 addition reduced MCL-1 protein levels in cancer cells (Fig. 3d top). This puts forth a major role of ERK signaling in cancer cells protected by bCAFs. To investigate how effective this paracrine effect is when CAFs themselves are exposed to treatments, we used a co-culture model using CD90 expression of bCAFs to discriminate the two cell-types after treatment (Fig. 3e bottom) (detailed in Methods). The presence of CAFs protected cancer cells from ABT-737, and U0126 treatment negated this effect, without impacting on bCAFs viability (Fig. 3e top).

### MCL-1 is also important for bCAFs survival

**Fig. 4:**
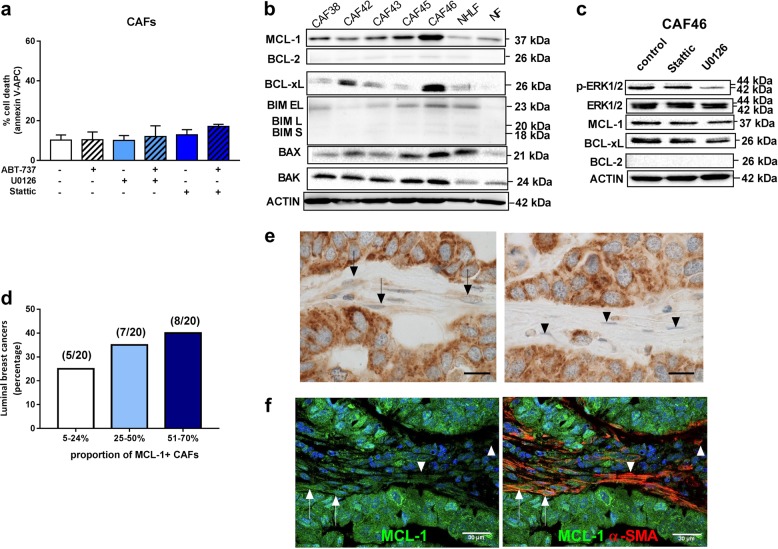
MCL-1 is highly expressed by bCAFs. CAFs (CAF42, CAF46, CAF65) were treated for 48 h with U0126 (5 µM) or Stattic (8 µM) alone or in combination with ABT-737 (1 µM). Percentage of positive Annexin-V-FITC apoptotic cells was measured by flow cytometry. **b** Anti-apoptotic (BCL-xL, BCL-2, MCL-1) and pro-apoptotic (BAX, BAK) proteins expression levels in normal human lung fibroblast (NHFL) and primary culture of breast cancer-associated fibroblasts (CAF) were evaluated using western blots analysis. **c** MCL-1, P-ERK, and ERK expression levels were evaluated by western-blot in CAFs (CAF42, CAF46) treated for 24 h with Stattic (8 µM) or U0126 (5 µM). **d**–**f** MCL-1 expression in luminal-B breast cancer fibroblasts. **d** Proportion of MCL-1–positive cancer-associated fibroblasts in 20 luminal breast cancers. **e** Chromogenic detection (DAB) of Mcl-1 by immunohistochemical analysis of luminal breast cancers, representative cases. MCL-1–positive fibroblasts (arrows) with extended cytoplasm and hypochromatic nuclei, morphology of activated fibroblasts are shown on the left (left). Fibroblasts with undetectable MCL-1 expression (arrowheads) with smaller size and hyperchromatic nuclei, morphology of fibrocytes are shown on the right. Original magnification ×1000, bar = 20 micrometers. **f** MCL-1 (green) and a-SMA (red) fluorescent co-staining. Arrows and arrowheads indicate high and low MCL-1 expression in α-SMA positive cells respectively. Left: Green and Blue overlay, right, Green blue and red overlay

Arguably, the maintenance of CAFs viability during treatment contributes to the efficiency of their pro-tumoral effects as it relies, at least in part, on cytokine production. In agreement with the co-culture experiments reported above, the viability of isolated bCAFs grown ex vivo was not detectably altered by U0126 or Stattic, alone or in combination with ABT-737 (Fig. 4a). To understand this robustness, we analyzed BCL-2 family protein expression and function in bCAFs themselves.

Expression profiles of bCAFs were compared to these in the NHLF cell line and in one primary culture of breast normal fibroblasts, NF. bCAFs and NHLF uniformly expressed detectable levels of pro-apoptotic proteins BAX, BAK, and BIM. BCL-xL expression fluctuated and BCL-2 expression was barely detectable. Notably, bCAFs systematically expressed MCL-1 at a higher level than in normal fibroblasts (Fig. 4b). BCL-xL and MCL-1 expressions were slightly decreased by U0126 and Stattic treatments (Fig. 4c). Thus MCL-1 expression (and to la lesser extent, that of BCL-xL) characterize bCAFs. Importantly, MCL-1 expression in bCAFs was confirmed by immunostaining of 20 formalin fixed luminal breast cancer samples (Fig. 4d–f and Supplementary information).

**Fig. 5:**
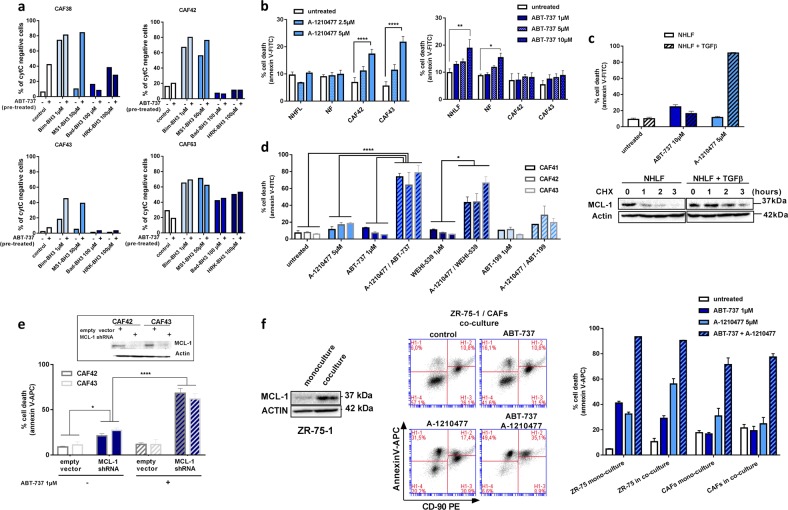
bCAFs rely on MCL-1 for their survival. **a** BH3 profiling. Values indicate the percentage of cytochrome c loss and are representative of one experiment. **b** NHLF, NF, and CAFs cells were treated with A-1210477 (2.5 or 5 µM) or ABT-737 (1, 5, or 10 µM) for 48 h in DMEM containing 0.5% FBS, apoptosis was measured by Annexin-V flow cytometry. **c** Top: NHLF pre-treated or not by TGFβ for 24 h were treated with 10 µM ABT-737 or 5 µM A-1210477 for 48 h in DMEM containing 0.5% FBS, apoptosis was measured by Annexin-V flow cytometry. Bottom: MCL-1 proteins expression levels in NHLF + /-TGF-β cells treated by cycloheximide for the indicated time, were evaluated using western-blot analysis. **d** CAFs were treated with 5 µM A-1210477 + /- ABT-737 1 µM, Wehi-539 1 µM or ABT-199 1 µM for 48 h in DMEM containing 0.5% FBS, apoptosis was measured by Annexin-V flow cytometry. **e** Percentage of apoptotic cells estimated in ABT-737-treated CAFs cells previously infected with the control vector or the MCL-1 sh-RNA for 72 h. MCL-1 protein expression in CAFs cells infected with the control vector or the MCL-1 sh-RNA was evaluated by western blotting (insert). **f** Co-culture experiments. ZR-75-1 cells (1/3) and CAFs (2/3) were treated for 48 h with ABT-737 1 µM and A-1210477 5 µM alone or in combination in DMEM 0.5% FBS. MCL-1 protein expression levels in ZR-75-1 cells isolated by fluorescence-activated cell sorting after their co-culture with CAF is shown on the left. Representative FACS profiles of cell death analyzes (using Annexin V-APC as a marker) in co-culture of ZR-75-1 cells and CAFs (marked by anti CD-90 PE) are shown in the middle. Percentage of apoptotic (Annexin-V–APC positive) tumor cells (CD9O negative) and CAFs (CD 90 positive) grown alone or in co-culture and treated with the indicated combination of BH3 mimetic, measured by flow cytometry are shown on the right. Data are means ± SEM from three independent experiments. *P*-value was determined by two-way ANOVA. *P < 0.05, **P < 0.01,, ****P < 0.0001

Functionally, BH3 profiling assays of bCAFs grown ex vivo ([19–21] detailed in Supplementary information) indicated that these cells: (i) respond to a BAX/BAK activating peptide (Bim-BH3); (ii) occasionally respond to a BCL-xL inhibitory peptides (Bad-BH3; HRK-BH3) or to a MCL-1 inhibitory peptide (MS1-BH3); (iii) systematically respond better to the latter peptide after their pre-treatment with ABT-737 (Fig. 5a). This indicates that bCAFs exhibit a mito-primed state and that MCL-1 plays a critical role to promote their survival together with ABT-737-sensitive BCL-xL.

bCAFs were treated with the MCL-1 specific inhibitor A-1210477 to further explore the role of MCL-1. This treatment, in contrast to treatment with ABT-737, induced significant cell death rates in itself over 48 h (Fig. 5b). It left intact normal breast fibroblasts NF or NHLF but the latter cells became sensitive following their “activation” by TGFβ (Fig. 5c, top) coinciding with MCL-1 protein stabilization in these cells (Fig. 5c, bottom). Cell death rates induced by A-1210477 were enhanced by co-treatment with ABT-737 and WEHI-539 but not with ABT-199, further supporting that BCL-xL activity contributes to the MCL-1 dependent viability of bCAFs (Fig. 5d). It should be noted that sensitivity to A-1210477 as a single agent was detected even in bCAFs that would not have necessarily been classified as MCL-1 dependent based on BH3 profiling assays. We assume that this apparent discrepancy stems from the fact that measuring the acute response to a BH3 peptide does not fully predict long-term effects of MCL-1 inhibition. In all cases, downregulation of MCL-1 by shRNA also induced cell death in bCAFs by itself, and sensitized them to ABT-737, indicating that the reported effects of A-1210477 are on-target (Fig. 5e).

To confirm that targeting MCL-1 would be beneficial by impacting both on bCAFs and on bCAFs influenced cancer cells, we used co-cultures of these two cell types challenged with BH3 mimetics as described above. We confirmed that MCL-1 expression in cancer cells was induced under these conditions (Fig. 5f, left). As shown in Fig. 5f (middle and right), addition of A-1210477 to ABT-737 led to dramatic cell death rates in each of the co-cultured cell type. Thus MCL-1 inhibition efficiently prevents the protective cross-talk between bCAFs and cancer cells.

### Co-variations between stromal score and MCL-1 expression characterize luminal breast cancers resistant to BCL-2 inhibition

Luminal breast cancers are highly heterogeneous and interactions between stromal and cancer cells are subject to high inter-tumor variations. To investigate how general might be the protective effects described above, we performed a retrospective analysis of a collection of primary ER-positive breast cancers. Freshly excised, treatment naïve specimen had been processed to be grown ex vivo as thick slices under culture conditions maintaining cell proliferation and viability during two days [22]. This had allowed us to expose to ABT-737 viable cancers cells in a preserved microenvironment and tissue architecture prior to fixation and banking, in parallel to another adjacent control slice of the same tumor that was left untreated.

**Fig. 6:**
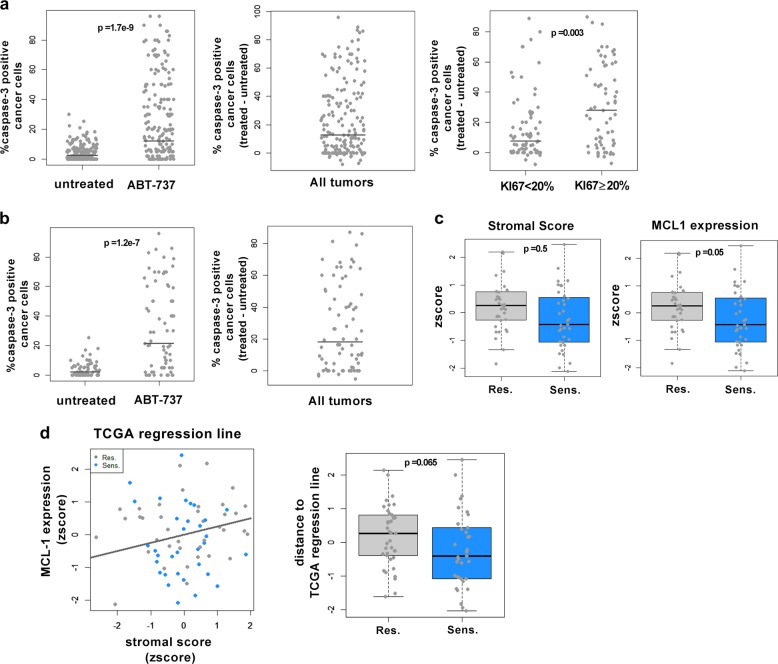
Co-variations between stromal score and MCL-1 expression characterize luminal breast cancers resistant to BCL-2 inhibition. **a** 169 ER^+^ primary human tumor samples were cultured 48 h with 1 μM ABT-737 or left untreated and then analyzed for active caspase-3 by immunohistochemistry. Left: data are represented as percentage of tumoral cells positive for active caspase-3 immunostaining in each treated and corresponding untreated specimen. Middle: percentage of tumoral cells positive for active caspase-3 immunostaining induced by ABT-737 above control (ABT-737 treated- untreated slice) is represented for all samples. Right: percentage of tumoral cells positive for active caspase-3 immunostaining induced by ABT-737 above control (ABT-737 treated- untreated slice) depending on the percentage of KI67 labelling as indicated. P-value were calculated using Wilcoxon test. **b** Left and Right: Data from the 71 samples used for molecular analysis are shown as in Fig. 5a Left and Right respectively. **c** Stromal score (*z*-score, Left) and MCL1 expression (*z*-score, Right) in “resistant” and “sensitive” groups (see Text for further details). *P*-value was calculated using Wilcoxon test. **d** Left: Plot of the 71 affymetrix samples with respect to their MCL1 mRNA expression and Stromal score (*z*-score) to which was added the TCGA regression line inferred from data used in Table 1. “Resistant” and “sensitive” groups are colored in gray and blue respectively. Right: Differences between MCL1 expression expected from the TCGA regression line and MCL1 expression samples from each group are presented as boxplots

We performed immunohistochemical analysis of 169 pairs of samples to evaluate the percentage of tumor cells that stained positive with an anti-active caspase-3 antibody, as a marker of apoptosis, in each of the two matched untreated and ABT-737 treated slices. ER-positive tumors, showed apoptotic responses to ABT-737 that ranged from non-detectable to major cell death rates above control (Fig. 6a, Left and Middle). Notably, tumors with a high proliferation rate (Ki67 ≥ 20, a feature that tends to associate with the aggressive LUM B subtype) significantly displayed a better sensitivity to ABT-737 induced apoptosis (Fig. 6a, Right).

Affymetrix expression data of 71 of the (untreated) corresponding tumors were available to study the relationship between stromal presence, MCL-1 expression and sensitivity to ABT-737 in luminal cancers (Fig. 6b, Left). We performed supervised analysis ranking tumor response based on a cutoff defined by the median cell death rates observed in these samples (19,5% difference between ABT-737 treatment and untreated control, Fig. 6b, Right). The so-called “resistant” samples (*n* = 35) exhibited stromal scores that were not statistically distinct from those from “sensitive” samples (*n* = 36) but MCL-1 levels were significantly higher in the “resistant” group” compared to the “sensitive” one (Fig. 6c). We plotted MCL-1 expression and stromal score of our 71 samples with respect to the regression line inferred from the TCGA data (used to characterize correlations shown in Table 1) (Fig. 6d, Left). In sensitive tumors, MCL-1 levels were further below levels expected from their stromal score than in the resistant ones (Fig. 6d, Right), indicating that sensitivity and low MCL-1 expression coincides with a weak participation of the stroma to the latter.

Thus resistance to induction of cell death by BCL-2 targeting, is regularly, albeit not systematically, found across freshly excised luminal breast cancers and expression data are consistent with the notion that the BCL-2 dependency of an individual tumor is mitigated, at least in part, by the contribution of the stroma to MCL-1 expression.

## Discussion

The molecular features of malignant cells that predominantly compose a breast cancer are critical not only to determine its molecular subtype but also to guide therapeutic choice. The proposition to use BCL-2 inhibitors as part of the armamentarium of luminal breast cancers follows this logic, as BCL-2 expression is intrinsically associated to estrogen receptor positivity. Preclinical studies validated the use of BCL-2 inhibitors to enforce tumor cell death in human luminal breast cancer [7] justifying a Phase 1b Study of Bcl-2 inhibition with ABT-199 in combination with tamoxifen in metastatic ER-positive breast cancers (ISRCTN98335443). The data we present here do not dispute the usefulness of BCL-2 inhibitors and in fact our ex vivo assays showing positive responses samples to prototypical ABT-737 in subsets of primary tumors strengthen the case. They establish, however, that the effects of such compounds can be mitigated by a stromal contribution to the expression and stability of the complementary anti-apoptotic protein MCL-1, which becomes a major therapeutic target under these circumstances. Evidence supporting the therapeutic targeting of MCL-1 was brought in the triple-negative breast cancer [23–25] and HER2-amplified molecular subtypes [16, 26]. Our study widens the therapeutic indications of MCL-1 inhibitors to luminal breast cancers. In this case, MCL-1 dependency of cancers cells is not intrinsically driven but induced by CAFs from the microenvironment. It would have been overlooked using experimental paradigms that do not take into account the multi-cellularity of breast tumors. This underscores the multifactorial nature of cancer cell mito-priming and the resulting difficulty to anticipate it from the pre-treatment molecular profile of a tumor. We propose that analysis, in bulk tumor expression data, of co-variations between molecular features of malignant cells and micro-environmental characteristics are more informative than differential gene expression per se.

Our data unambiguously attribute to bCAFs an anti-apoptotic effect on luminal breast cancer cells, which relies on paracrine and autocrine functions of IL-6. Serum IL-6 levels are high in the subjects with ductal carcinoma advanced stage [27] and aberrantly elevated IL-6 is associated with a poor prognosis in breast cancer [27]. The autocrine function we ascribe to IL-6: (i) is consistent with the fact that high expression of IL-6 receptor in spindle-shaped stromal cells associates with shorter disease-free survival, metastasis-free survival, and overall survival in breast cancers [28]; (ii) is consistent with a role of IL-6 in maintaining CAF activation [29]; (iii) evokes the reported role of autocrine, CAFs derived IL-6 in endocrine resistance [11, 30]. This function implies that other secreted factors, yet to be identified, play a more direct role in the apoptosis resistance induced by stromal cells. Paracrine IL-6, possibly together with these factors, favor activation of the MEK signaling pathway in cancer cells. Because this pathway has well established positive effects on MCL-1 expression and survival [31], we infer it exerts context dependent MCL-1 mediated survival effects in luminal breast cancers.

As biologically active CAFs produce complex overlapping signals to protect cancer cells, their survival maintenance during the course of treatment might be particularly problematic. Our identification of specific vulnerabilities in bCAFs, implying that BH3 mimetics might target them, is thus of particular importance: it offers the prospect of directly eradicating these stromal cells. The “mito-primed” state of bCAFs evokes a similar, pharmacologically exploitable, state in fibrotic fibroblasts, that share common features with bCAFs such as αSMA expression [32]. Fibrotic fibroblasts depend, however, on BCL-xL for survival while bCAFs also express MCL-1 and rely on it for survival. Of note, we observed that MCL-1–positive breast cancer fibroblasts tended to have an activated morphology, whereas MCL-1–negative CAFs tended to resemble fibrocytes, suggesting a specific role for MCL-1 that needs to be determined. Intriguingly, Gores et al. [33], showed that Navitoclax was sufficient to induce apoptosis in human CAFs from cholangiocarcinoma, implying that mito-priming in CAFs has to be explored in an organ-specific manner.

In sum, luminal breast cancer cells and CAFs in symbiosis recruit specific BCL-2 family members to promote their survival. This advocates for BH3 mimetic based targeted apoptosis as an option to prevent the pro-tumoral interactions between these two cell types, both vulnerable to these compounds. In particular, MCL-1 inhibition, which is now attainable clinically as a potent BH3 inhibitor was recently characterized and shown to be tolerable [34], might be particularly appropriate as it should counteract the effect of a pathological stroma on cancer cells in the same time as it should affect stromal cells that produce this effect. In the light of the high heterogeneity of bCAFs themselves [35], more thorough biological exploration of these cell populations will help refine and ameliorate this promising approach.

## Materials and methods

### Human cancer-associated fibroblast isolation

Fresh human mammary samples were obtained from treatment-naive patients with invasive carcinoma after surgical resection at the Institut de Cancérologie de l’Ouest, Nantes/Angers, France. As required by the French Committee for the Protection of Human Subjects, informed consent was obtained from enrolled patients and protocol was approved by Ministère de la Recherche (agreement n°: DC-2012-1598) and by local ethic committee (agreement n°: CB 2012/06). To isolate breast cancer-associated fibroblasts (bCAFs), breast tissues were minced and enzymatically digested with a mixture of collagenase I (1 mg/ml Sigma) and hyaluronidase (1 mg/ml Sigma) in Dulbecco’s Modified Eagle Medium (DMEM Thermo Fisher Scientific) supplemented with 10% FBS, 2 mM glutamine and 1% Penicillin/Streptomycin, using a rotator for approximately four hours at 37 °C. Post-digestion, the tissues (isolated cells and partially digested-tissues) were washed and cultured in the same media at 37 °C. All the fibroblasts used in the experiments were at early passage (between three and seven). Normal Fibroblast (NF) were obtained from mammary reconstruction, using the same protocol.

### In vitro culture assays

The human breast cancer cell lines ZR-75-1, and T47D were purchased from American Type Culture Collection (Bethesda, MD, USA) and were cultured using media according to manufacturers’ protocols. The normal human lung fibroblast (NHLF) cell line were purchased from Lonza, and were cultured in DMEM supplemented with 3% FBS, 2 mM Glutamine and 1% Penicillin/Streptomycin.

Co-culture is based on the combined culture of 1:3 mix of breast cancer cells (ZR-75-1) and CAFs in monolayer in DMEM 10% FBS, 2 mM Glutamine and 1% Penicillin/streptomycin. After 24 h, the co-culture was maintained in DMEM 1% FBS for 48 h, then treated as indicated for additional 48 h. In this case, monoculture assays (ZR-75-1 or CAFs) were performed in the same conditions.

For the preparation of Fibroblasts-conditioned media, culture of 6 × 10^5^ cells in 8 ml of DMEM without FBS were used. The shRNA infected CAFs CM were made from the culture of 2 × 10^5^ cells in 2.5 ml of DMEM without FBS, and treated or not with IL-6 recombinant (500 pg/ml). 48 h later, CM were collected, centrifuged (1500 rpm, 4 min) and supplemented with 0.5% FBS prior to incubation with cancer cells.

RNA interference was performed by lentiviral transduction as previously described [36]. The following shRNA sequence was used in lentiviral experiments targeting BCL-X_L_: 5′-AGGATACAGCTGGAGTCAG-3′; IL-6 5′-GGAGACATGTAACAAGAGT-3′; MCL-1: 5′-GAATGCCAGTGACCTGTGT-3′

Immunocytochemistry, treatments, ELISA assays, Immuno-blot analysis, RNA isolation, and quantitative real-time PCR and Cell death assays are detailed in Supplementary Methods.

### Histological experiments

Fresh human mammary samples from treatment-naive patients were collected after surgical resection at the Institut de Cancérologie de l′Ouest, René Gauducheau, Nantes, France, between 2009 and 2017 according to the same procedure and protocol approval as reported above. Immunohistochemical analysis on treatment naïve specimen grown ex vivo as thick slices for two days prior fixation was performed as previously described [22]. Active caspase-3 immunostaining were assessed to determine the percentage of labeled cells in at least 200 carcinomatous cells counted. Non-neoplastic cells were excluded from counting.

For investigation of MCL-1 expression in clinical samples of breast cancers, immunostaining was performed using 3-µm-thick tissue sections of formalin-fixed, paraffin-embedded (FFPE) breast cancers, diagnosed at the Institut de Cancérologie de l′Ouest Nantes/Angers, France, as detailed in Supplementary Methods.

### Analysis of mRNA expression data in tumor samples

Gene expression analysis was performed on 81 breast tumor samples using Affymetrix® Human Genome U133 Plus 2.0 Arrays (Affymetrix®, Santa Clara, CA, USA) measuring over 43,000 transcripts representing over 20,000 human genes [37]. In our study, we retain the 71 ER-positive breast tumor samples.

mRNA expression established in the Breast Invasive Carcinoma study (TCGA, Nature 2012 [5]) were downloaded from the TCGA data portal (https://tcga-data.nci.nih.gov/docs/publications/brca_2012/). Stromal scores of 488 of these tumor samples originate from Estimate data [34–38] downloaded from the bioinformatics website of the MD Anderson Cancer Center (http://bioinformatics.mdanderson.org/estimate/disease.html, Disease Type: breast cancer, Platform Type: AGILENT G4502A). Correlations between Stromal Score and corresponding mRNA expression levels were performed and plotted using R program. Estimate package (http://bioinformatics.mdanderson.org/estimate/rpackage.html) was used to compute stromal score from the affymetrix microarray expression dataset of the 71 samples of our breast cancer tumor collection.

### Statistical analysis

Two-way analysis of variance (ANOVA) was used for statistical analysis for overall condition effects with GraphPad Prism 5.0 Software. All data are presented as mean ± SEM of at least three independent experiments. The symbols correspond to a *P*-value inferior to *0.05, **0.01, ***0.001 and ****0.0001.

## Supplementary information

Supplementary Information

Supplementary Methods

Supplementary Legends

Supplementary Table 1

Supplementary Table 2

Supplementary Figure 1

Supplementary Figure 2

Supplementary Figure 3
