# Supplementary material for: Interactions between cancer-associated fibroblasts and tumor cells promote MCL-1 dependency in estrogen receptor-positive breast cancers
Source: Oncogene. 2019 Jan 10;38(17):3261–73. doi: 10.1038/s41388-018-0635-z (PMC6756023; doi:10.1038/s41388-018-0635-z)
Supplement: Supplementary file 1 — Supplementary Information [file 41388_2018_635_MOESM1_ESM.docx]

**SUPPLEMENTARY INFORMATION**

**Characterization of primary breast cancer associated CAFs**

To confirm that we successfully isolated essentially pure populations of bCAFs, we checked that the resulting cells showed a typically fibroblastic phenotype (Fig. 1a), expressed significantly higher levels of specific mRNAs (alpha-smooth muscle actin-α-sma; vimentin; fibronectin; tenascin C; FGF2; IL-6 ^35^) compared to luminal breast cancer cell lines (T47-D and ZR-75-1) (Supplementary Fig. 1a), and do not expressed detectable cytokeratin (Fig. 1b). Activated phenotype of CAFs was confirmed by expression of the two markers, α-SMA and FAP in these cells compared to normal primary fibroblasts NF (Fig.1C left and Fig.S1b). We confirmed that collagen contractility, a characteristic of activated fibroblasts, was enforced in CAFs *versus* NF (Fig. 1C, left).

**Biochemical characterization of protective factors presented in bCAFs derived CM**

In an attempt to document the nature of the secreted factors implicated in CAFs protective effects, conditioned media were heat inactivated by boiling for 10 min prior to use it. The boiled conditioned media no longer has effect on ABT-737 induced cancer cells apoptosis suggesting a significant role of proteins in this effect (Figure S2a). Furthermore, we found that the protective factor(s) is (are) contained in the soluble fraction of the conditioned media and not in the extracellular vesicle fraction (Fig. S2b) and we confirmed its thermo-sensitivity (Fig. S2c).

**Immunocytochemistry**

Cells were fixed in PBS containing 4% paraformaldehyde/4% sucrose for 15 min. Cells were permeabilized (except for FAP labelling) for 5 min at room temperature in 0.25% Triton-X-100 in PBS, washed twice with PBS, and incubated for 30 min at 37°C in PBS containing 10% BSA. Cells were incubated overnight at 4°C with primary antibodies diluted in PBS containing 3% BSA. Antibodies used were as follows: mouse anti pan-cytokeratin (1:100, Abcam, Cambridge, MA, USA), mouse anti alpha-SMA (1:300, Abcam, Cambridge, MA, USA) and mouse anti FAP (1:200 Abcam, Cambridge, MA, USA). After washing, cells were incubated for 90 min at room temperature with the appropriate Alexa 488-conjugated secondary antibodies diluted in PBS containing 3% BSA. Cells were washed with PBS and mounted with ProLong Diamond Antifade Reagent with DAPI (Invitrogen).

**Correlation analysis in TCGA data sets**

We used cBioportal to explore the established expression data of 321 luminal breast cancers in the Breast Invasive Carcinoma study (TCGA ^36^). We identified 518 genes whose mRNA expression positively correlated with that of MCL-1 (with a Pearson score >3) (not shown) and 16 of them where typed as coding for cytokines (Supplementary Table 2).

**Immunohistochemistry analysis of bCAFs from formalin fixed, paraffin embedded human breast cancer samples**

This analysis showed MCL-1 expression in CAFs and also in the cytoplasm of breast carcinoma cells, some tumor-infiltrating lymphocytes, and most endothelial cells. The proportion of MCL-1–positive CAFs ranged from 5 to 70% (mean 43 ± 23%) with MCL-1–positive CAFs outnumbering MCL-1–negative ones in 8/20 luminal breast cancers (Fig. 3d). MCL-1–positive breast cancer fibroblasts tended to have an activated morphology, with larger size, extended cytoplasm, and hypochromatic nuclei (Fig. 4e, left), whereas MCL-1–negative CAFs tended to resemble fibrocytes, with smaller size, barely visible cytoplasm, and hyperchromatic nucleus (Fig. 4e, right). These observations were confirmed by immunofluorescent co-staining of MCL-1 and a fibroblastic marker, a-SMA (Fig. 4f).

**BH3 profiling assays**

These assays were performed to investigate the propensity of bCAFs to undergo mitochondrial outer membrane permeabilization (MOMP), and to characterize the identity of anti-apoptotic protein(s) involved in maintaining mitochondrial integrity. For this purpose, permeabilized CAFs (pre-treated or not with ABT-737 as indicated) were incubated with a range of BH3 peptides that are functionally distinct as they interact with differing BCL-2 family members (BAX/BAK or selective subsets of anti-apoptotic proteins). Cytochrome c release (measured by FACS) was used as a readout for loss of mitochondrial permeability (see Supplementary Methods). The following peptide were used:

- a BAX/BAK activating peptide derived from the sequence of BIM (BIM-BH3 to probe the cells ability to undergo MOMP)
- a BCL-2/BCL-xL inhibitory peptide (BAD-BH3)
- a BCL-xL inhibitory peptide (HRK-BH3)
- a MCL-1 inhibitory peptide (MS1, derived from an optimized NOXA-BH3 sequence)
