# Supplementary material for: Interactions between cancer-associated fibroblasts and tumor cells promote MCL-1 dependency in estrogen receptor-positive breast cancers
Source: Oncogene. 2019 Jan 10;38(17):3261–73. doi: 10.1038/s41388-018-0635-z (PMC6756023; doi:10.1038/s41388-018-0635-z)
Supplement: Supplementary file 2 — Supplementary Methods [file 41388_2018_635_MOESM2_ESM.docx]

**SUPPLEMENTARY METHODS**

**Treatments**

BH3 mimetics, ABT-737, ABT-199, A-1210477 (Selleckchem, Houston, TX, USA), WEHI-539 (Euromedex, Strasbourg, France), and cell signaling pathways inhibitors U0126 (Invivogen, San Diego, CA,
USA ), Stattic (Selleckchem), Doxorubicin (G-GAM Laboratories, Creteil, France), 5-FluoroUracil (Sigma, St Louis, MO, USA), Cisplatin (TEVA laboratories, Courbevoie, France) were dissolved in DMSO at the indicated concentrations. Trastuzumab (Herceptin) and fulvestrant (Falsodex) were purchased from Roche (Basel, Switzerland) and Astrazeneca (London, England), respectively. Recombinant IL-6 (PeproTech, Rocky Hill, NJ, USA) was dissolved in H_2_0 0.1% BSA at 0.5 to 10 µg/mL and added to cell media one hour prior to treatment. TGFβ (R&D Systems, Minneapolis, MN, USA) was dissolved in DMSO and added to NHLF at 2 ng/ml for 24 hours prior to treatment.

**Collagen gel contraction assay**

Fibroblasts (100 000 cells) were re-suspended in 500 μl collagen suspension (1 mg/ml) in DMEM supplemented with 5% FBS. Cell suspension was cast into each well of 24-well tissue culture plate and incubated at 37°C for 1 h in order to facilitate gelation. Following this, gels were released from the surface of the culture well using a sterile tip. Contraction between the different conditions was evaluated at 3 h.

**Extracellular vesicles isolation**

The conditioned medium was centrifuged at 2.000 x g for 15 min (pellet 2K) and 10.000 x g for 30 min at 4°C (pellet 10K) to remove the dead cells and debris. The supernatant was centrifuged at 100.000 x g for two hours to obtain the extracellular vesicle pellet (100K supernatant). The pellet containing extracellular vesicle was diluted with filtered phosphate-buffered saline (PBS) and centrifuged at 100.000 x g for two hours (pellet 100K). The pellets obtained (2K, 10K and 100K) were resuspended in DMEM with 0,5 % FBS. The pellets and supernatant (100K) are used like conditioned medium on cancer cells, and then cancer cells were treated to ABT-737 (1 µM) for 48 h.

**ELISA assays**

Levels of IL-6 in conditioned-media were determined by ELISA. The culture medium of CAFs silencing or not for IL-6, CAFs treated with Stattic 8µM for 48 hours, NHLF, NHLF + TGFβ and ZR-75-1 cells were harvested and IL-6 was detected according to the manufacturer’s protocol (ebioscience, San Jose, CA, USA). Three independent experiments were performed.

**Immunoblot analysis**

Cells were resuspended in lysis buffer (1% SDS; 10 mM EDTA; 50 mM Tris-Hcl pH 8.1; 1 mM PMSF; 10 μg/ml^−1^ aprotinin; 10 μg/ml^−1^ leupeptin; 10 μg/ml^−1^ pepstatin; 1 mM Na3VO4 and 50 mM NaF) and sonicated with Bioruptor apparatus from Diagenode. For western blotting, following SDS–PAGE, proteins were transferred to 0.45 µM nitrocellulose membranes using Trans-Blot® Turbo™ Transfer System Cell system (Bio-Rad). The membrane was then blocked in 10% nonfat dry milk TBS 0.1% Tween 20 and incubated with primary antibody overnight at 4°C. Blots were incubated with the appropriate secondary antibodies for 1 h at room temperature and visualized using the Chemi-Doc XRS+ system (Bio-Rad). Primary antibodies used were anti-BCL-X_L_ (abcam, ab32370), anti-MCL-1 (Santa Cruz, sc-819), anti-BCL-2 (Dako, M0887), anti-Bax (Dako, A3533), anti-Bak (Cell signaling, 3814), anti-BIM (Millipore, AB17003), anti-p-ERK T202/Y204 (Cell Signaling, 4370), anti-ERK (Cell Signaling, 9102) anti-STAT-3 (BD transduction Laboratories, 610190), anti p-STAT-3 Y705 (Cell Signaling, 9145L) and anti-β-ACTIN (Millipore, MAB1501R).

**Apoptosis assays**

Cell death was assessed by an Annexin-V FITC binding assay (Miltenyi) performed according to manufacturer's instructions. Flow-cytometry analysis was performed on a Accuri C6 flow cytometer from BD biosciences. For co-culture apoptosis assay, cell suspension was stained with PE-conjugated human CD90 antibodies (BD Biosciences, 555596) prior to Annexin-V APC (BD Biosciences,550474) binding assay according to the manufacturer’s instructions.

**Cytochrome C release**

Briefly, cells were fixed and permealized using FIX & PERM Cell Fixation and Permeabilization Kits (ebioscience, San Jose, CA, USA). Cell suspension was stained with Alexa647-conjugated human cyt-C antibody (BD Biosciences, 560263).

**Caspase 9 activity**

The activity of caspase-9 was measured by Caspase-Glo-9 assay kit according to the manufacturer's instructions (Promega).

**RNA isolation and quantitative real-time PCR**

Total RNA was isolated using Nucleospin RNA (Macherey-nagel, Hoerdt, France) and transcribed into cDNA by Maxima First Strand cDNA synthesis Kit (Thermo scientific). Quantitative RT-PCR (qPCR) was performed using the EurobioGreen qPCR Mix Lo-Rox with qTOWER (Analityk-jena, jena, Germany). Reaction was done in 10 μl final with 4 ng RNA equivalent of cDNA and 150 nM primers. Relative quantity of mRNA was estimated by Pfaffl method (Pfaffl *et al.*, Nucleic Acids Res, 2001) and normalized on the average relative quantity of three housekeeping genes.

ACTB 5'-AGAAAATCTGGCACCACACC / CAGAGGCGTACAGGGATAGC-3'

ACTA2 5’ TATCCCCGGGACTAAGACGG/ TACAGAGCCCAGAGCCATTG-3’

B2M 5'-CGTGGCCTTAGCTGTGC / AATGTCGGATGGATGAAACC-3'

FGF2 5’- CTTCCTGCGCATCCACCCCG/ AGCCAGGTAACGGTTAGCACACA-3’

FN1 5’-AGAAGTGGTCCCTCGGCCCC/ GGGTTACCAGTTGGGGAAGCTCG-3’

GAPDH 5'-CAAAAGGGTCATCATCTCTGC / AGTTGTCATGGATGACCTTGG-3'

IL6 5’- AAGATGTAGCCGCCCCACACA/ CTGCCAGTGCCTCTTTGCTGCT-3’

MCL-1 5'-TCGGTACCTTCGGGAGCAGGC / CCCAGTTTGTTACGCCGTCGCT-3'

PDGFRB 5’-AACTGTGCCCACACCAGAAG / CAGGAGAGACAGCAACAGCA-3’

RPLP0 5'-AACCCAGCTCTGGAGAAACT / CCCCTGGAGATTTTAGTGGT-3'

TENC 5’- CCAAAACCATCAGTGCCACA/ AAACTGTGAACCCGTAGGGA-3’

VIM 5’-GAGAACTTTGCCGTTGAAGC/ TCCAGCAGCTTCCTGTAGGT-3’

**Intracellular BH3 profiling**

BH3 profiling was performed as previously described^37^. Briefly, cells were resuspended in DTEB buffer^38^. 100 000 cells in DTEB buffer were added to twice the final concentration of each peptide treatment (BIM, BAD, HRK, NOXA#) diluted in 0.002% digitonin/DTEB buffer. BH3 profiling was performed using the BIM BH3 (1 and 10 μM), BAD BH3 (100 μM) and HRK BH3 (100 μM) peptides, and NOXA# BH3 (100 and 50 μM) peptides. Peptide sequence for NOXA (MS1 named NOXA#) has been previously described^39^. Mitochondria of permeabilized cells were exposed to peptides for 45 min at 27°C before fixation with 2% formaldehyde at room temperature for 15 min. After addition of neutralizing buffer (Tris 0.41 mol/L glycine pH 9.1) for 5 min, cells are stained with anti-cytochrome *c*–Alexa647 (BLE612310, Ozyme) 1:40 in 0.1% Saponin/1%BSA/PBS overnight at 4°C. Loss of cytochrome c was analyzed by a flow cytometer (FacsCalibur). The quantification loss induced by each peptide was analyzed by gating the cytochrome c negative population.

**Immunocytochemistry**

Briefly cells were grown on coverslip glass. Cells were fixed with paraformaldehyde 4%/sucrose 4%, permeabilized with 0.25% Triton X-100 before blocking (BSA 10% in PBS) and immunostaining with specific antibodies diluted in 3% BSA/PBS; mouse anti alpha-SMA (1:300, Abcam, AB119952), rabbit anti FAP (1:200, Abcam, AB53066) and rabbit anti-pan cytokeratin (1:400, Abcam, AB217916).

**Immunohistochemistry**

Chromogenic immunohistochemistry was run in a Ventana BenchMark XT immunostainer (Ventana Medical Systems, Tucson, AZ, USA). Sections were dried at 60°C for 2 hours, deparaffinized, pretreated at 95°C for 32 minutes in a basic buffer (CC1, Cell Conditioning Medium-1, pH 8.4, Ventana Medical Systems) for antigen retrieval, and stained for 32 minutes at 37°C with anti-Mcl-1 rabbit polyclonal antibody (Santa Cruz Biotechnology, sc-819) at 0.8 µg/ml in an Antibody Diluent (Ventana Medical Systems). Chromogenic detection was performed using the Ventana OptiView DAB IHC detection kit, followed by counterstaining with one drop of hematoxylin-II for four minutes and one drop of bluing reagent for 4 minutes. Slides were then removed from the immunostainer, washed in water with a drop of dishwashing detergent, and mounted. Negative controls were obtained by replacement of the primary antibody with normal rabbit serum (Negative Control Rabbit Ig, 10 µg/ml, Ventana Medical Systems). For fluorescent immunohistochemistry, briefly sections were deparaffinized, incubated in a Decloaking Chamber™ (Biocare Medical) (110°C for 90s in 10 mM Tris/1mM EDTA pH9 solution) for heat-induced epitope retrieval, and stained over-night at 4°C with anti-Mcl-1 rabbit polyclonal antibody (Santa Cruz Biotechnology, sc-819) 1/50 and mouse anti alpha-SMA (1:300, Abcam, AB119952) in an antibody diluent (Dako). After washing, sections were incubated for 60 min at room temperature with the appropriate secondary antibodies and mounted with ProLong Diamond Antifade Reagent with DAPI (Invitrogen).
