# Supplementary material for: Interactions between cancer-associated fibroblasts and tumor cells promote MCL-1 dependency in estrogen receptor-positive breast cancers
Source: Oncogene. 2019 Jan 10;38(17):3261–73. doi: 10.1038/s41388-018-0635-z (PMC6756023; doi:10.1038/s41388-018-0635-z)
Supplement: Supplementary file 3 — Supplementary Legends [file 41388_2018_635_MOESM3_ESM.docx]

**SUPPLEMENTARY LEGENDS**

**Figure S1.**  **(a)** mRNA expression level of fibroblasts markers measured by quantitative RT-PCR in breast cancer lines (ZR-75-1 and T47-D) and a panel of primary culture of CAFs. Arbitrary unit of relative quantity of mRNA normalized to the mean of RPLP0, B2M and GAPDH relative expression. **(b)** α-smooth muscle actin (α-SMA) and Fibroblast activation protein (FAP) proteins expression levels in primary culture of normal fibroblast (NF) and primary culture of breast cancer associated fibroblasts (CAF) were evaluated by western blots analysis. **(c)** mRNA expression level of PDGFRB measured by quantitative RT-PCR in NHLF treated or not with TGF-β 2nM for 48 hours. Arbitrary unit of relative quantity of mRNA normalized to the mean of RPLP0, B2M and GAPDH relative expression. **(d, e, g, h)** ZR-75-1 cells were treated for 48 hours with (**c**) ABT-199 (10 µM) +/- Fulvestrant (1 µM), **(d)** ABT-199 (10 µM) +/- Doxorubicin (2.5 µM)/5-Fluorouracil (27.5 µM)/Cisplatin (55 µM), **(g)** ABT-737 (1 µM) +/- Herceptin (25 µg/ml) or **(h)** Herceptin (25 µg/ml) +/- Doxorubicin (2.5 µM)/5-Fluorouracil (27.5 µM)/Cisplatin (55 µM) in presence of non-conditioned media (Control) or media conditioned for 48h by Cancer Associated Fibroblasts (CAFs). Percentage of positive Annexin-V-FITC apoptotic cells was measured by flow cytometry. Anti-apoptotic (BCL-xL, BCL-2, MCL-1) proteins expression levels in ZR-75 treated by fulvestrant (**c**, right) or Herceptin (**g**, right) or in CAFs in the same conditions (**i**) were evaluated using western blots analysis. **(f)** ZR-75-1 cells were treated for 48 hours with WEHI539 (1, 5 or 10 µM). Percentage of positive Annexin-V-FITC apoptotic cells was measured by flow cytometry. Data are means ± SEM from three independent experiments. P value was determined by two-way ANOVA. *P<0.05, **P<0.01, ***P<0.001, ****P<0.0001

**Figure S2**. ZR-75-1 (**a, b, c**) or T-47D (**a**, right) cells were treated for 48 hours with ABT-737 (1 µM) in presence of non-conditioned media (non-CM) or **(a)** heat-denatured (or not) conditioned media (CM) from Cancer Associated Fibroblasts (CAFs), or (**b**) the different pellets or supernatant isolated from CAFs conditioned media (2K=2.000 x g; 10K=10.000 x g; 100K supernatant; 100K=100.000 x g), or **(c)** the 100K supernatant heated for 10 min at 95°C. Pellet was resuspended in the initial corresponding volume of media and, as previously, supplemented with 0.5% FBS. Percentage of positive Annexin-V-FITC apoptotic cells was measured by flow cytometry. Data are means ± SEM from three independent experiments. P value was determined by two-way ANOVA. ****P<0.0001, ns: not significant.

**Figure S3.** (**a**) Interleukin-6 concentration was measured by ELISA in conditioned media from ZR-75-1, T-47D, NHLF, TGFβ activated NHLF or CAFs. (**b**) ZR-75-1 and T-47D cells were treated for 48 hours with ABT-737 (1 µM) with or without recombinant IL-6 (0.5 or 1 ng/ml). Percentage of positive Annexin-V-FITC apoptotic cells was measured by flow cytometry. **(c, d, e)** Interleukin-6 concentration was measured by ELISA in conditioned media from bCAFs (c) +/-IL-6 shRNA, (d)treated for 24h with or without Stattic 8µM and (e) treated for 48h with or without the indicated targeted therapies. Data are means ± SEM from three independent experiments. P value was determined by two-way ANOVA. *P<0.05, **P<0.01

**Supplementary Table 1:** Clinical characteristics of primary breast tumors samples used for primary culture of bCAFs. ER estrogen receptor, PR progesterone receptor, HER2 human epidermal growth factor receptor 2, FISH fluorescence in situ hybridization

**Supplementary Table 2:** Cytokines whose mRNA expression positively correlated with that of MCL-1 (with a Pearson score >3).
