# Supplementary material for: Interactions between cancer-associated fibroblasts and tumor cells promote MCL-1 dependency in estrogen receptor-positive breast cancers
Source: Oncogene. 2019 Jan 10;38(17):3261–73. doi: 10.1038/s41388-018-0635-z (PMC6756023; doi:10.1038/s41388-018-0635-z)
Supplement: Supplementary file 4 — Supplementary Table 1 [file 41388_2018_635_MOESM4_ESM.docx]

|  | Patient's age (years) | Immunohistochemical score | | FISH | Tumor grade | Tumor size (mm) | histological type |
| --- | --- | --- | --- | --- | --- | --- | --- |
| CAFs |  |  |  |  |  |  |  |
|  |  | ER | PR | HER2 status |  |  |  |
| #038 | 74 | 100 | 0 | 1+ | III | 10 | Luminal B |
| #041 | 78 | 100 | 80 | 1+ | III | 40 | Luminal A |
| #042 | 62 | 100 | 8 | 2+NA | II | 23 | Luminal B |
| #043 | 42 | 100 | 25 | 1+ | III | 18 | Luminal A |
| #045 | 43 | 100 | 100 | 3+ | III | 15 | Luminal B like HER2 enriched |
| #046 | 38 | 95 | 80 | 3+ | III | 17 | Luminal B like HER2 enriched |
| #048 | 50 | 80 | 80 | 3+ | II | 20 | Luminal B like HER2 enriched |
| #049 | 78 | 95 | 70 | 2+ NA | II | 20 | Luminal B |
| #063 | 42 | 95 | 95 | 1+ | II | 30 | Luminal A |

Supplementary Table 1

Clinical characteristics of breast tumors samples used for primary culture of bCAFs
