# Supplementary material for: Interactions between cancer-associated fibroblasts and tumor cells promote MCL-1 dependency in estrogen receptor-positive breast cancers
Source: Oncogene. 2019 Jan 10;38(17):3261–73. doi: 10.1038/s41388-018-0635-z (PMC6756023; doi:10.1038/s41388-018-0635-z)
Supplement: Supplementary file 5 — Supplementary Table 2 [file 41388_2018_635_MOESM5_ESM.docx]

| Pearson  coefficient | GENE | Cytokine |
| --- | --- | --- |
| 0.390 | LIF | LIF, interleukin 6 family cytokine |
| 0.380 | IL18 | interleukin 18 |
| 0.370 | CXCL2 | C-X-C motif chemokine ligand 2 |
| 0.350 | OSM | oncostatin M |
| 0.340 | CXCL16 | C-X-C motif chemokine ligand 16 |
| 0.340 | CXCL17 | C-X-C motif chemokine ligand 17 |
| 0.340 | IL15 | interleukin 15 |
| 0.340 | TNFSF10 | TNF superfamily member 10 |
| 0.330 | IL6 | interleukin 6 |
| 0.320 | CCL23 | C-C motif chemokine ligand 23 |
| 0.320 | IL7 | interleukin 7 |
| 0.310 | CMTM3 | CKLF like MARVEL transmembrane domain containing 3 |
| 0.310 | CXCL1 | C-X-C motif chemokine ligand 1 |
| 0.310 | IL12A | interleukin 12A |
| 0.310 | NAMPT | nicotinamide phosphoribosyltransferase |
| 0.310 | XCL2 | X-C motif chemokine ligand 2 |

Supplementary Table 2
