# Supplementary figures and images for: Interactions between cancer-associated fibroblasts and tumor cells promote MCL-1 dependency in estrogen receptor-positive breast cancers
Source: Oncogene. 2019 Jan 10;38(17):3261–73. doi: 10.1038/s41388-018-0635-z (PMC6756023; doi:10.1038/s41388-018-0635-z)

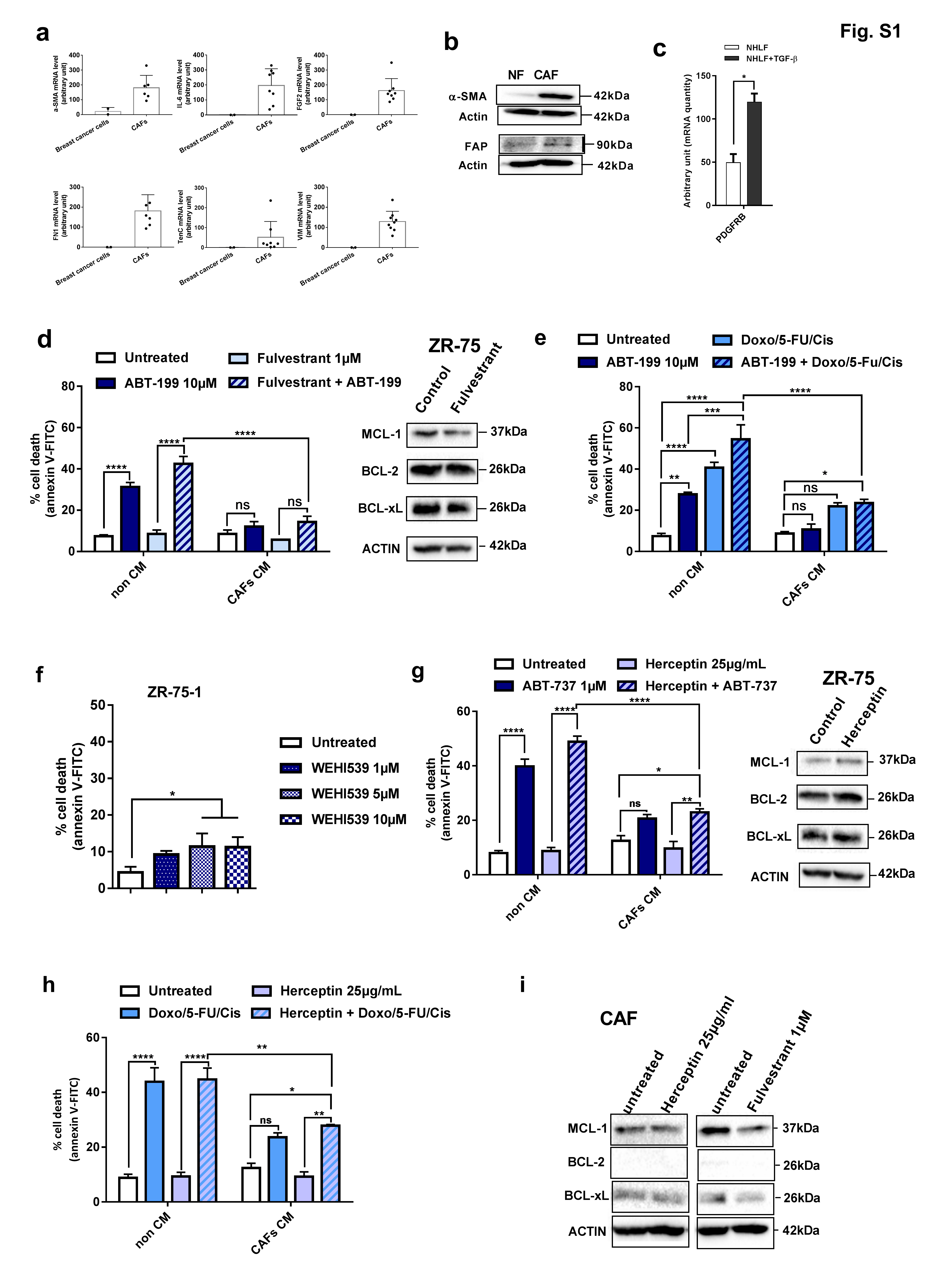

Supplement: Supplementary file 6 — Supplementary Figure 1 [file 41388_2018_635_MOESM6_ESM.tif]

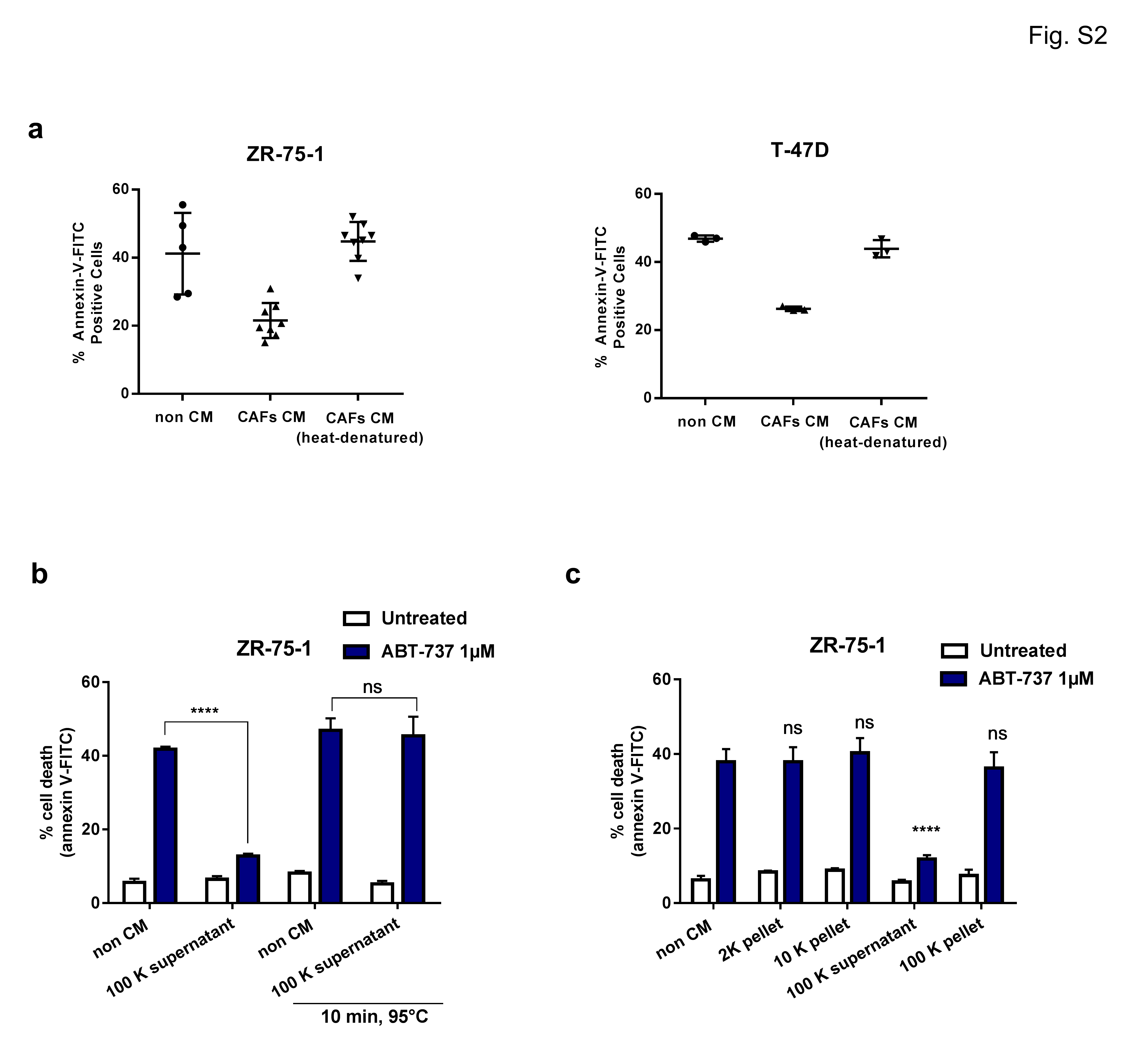

Supplement: Supplementary file 7 — Supplementary Figure 2 [file 41388_2018_635_MOESM7_ESM.tif]

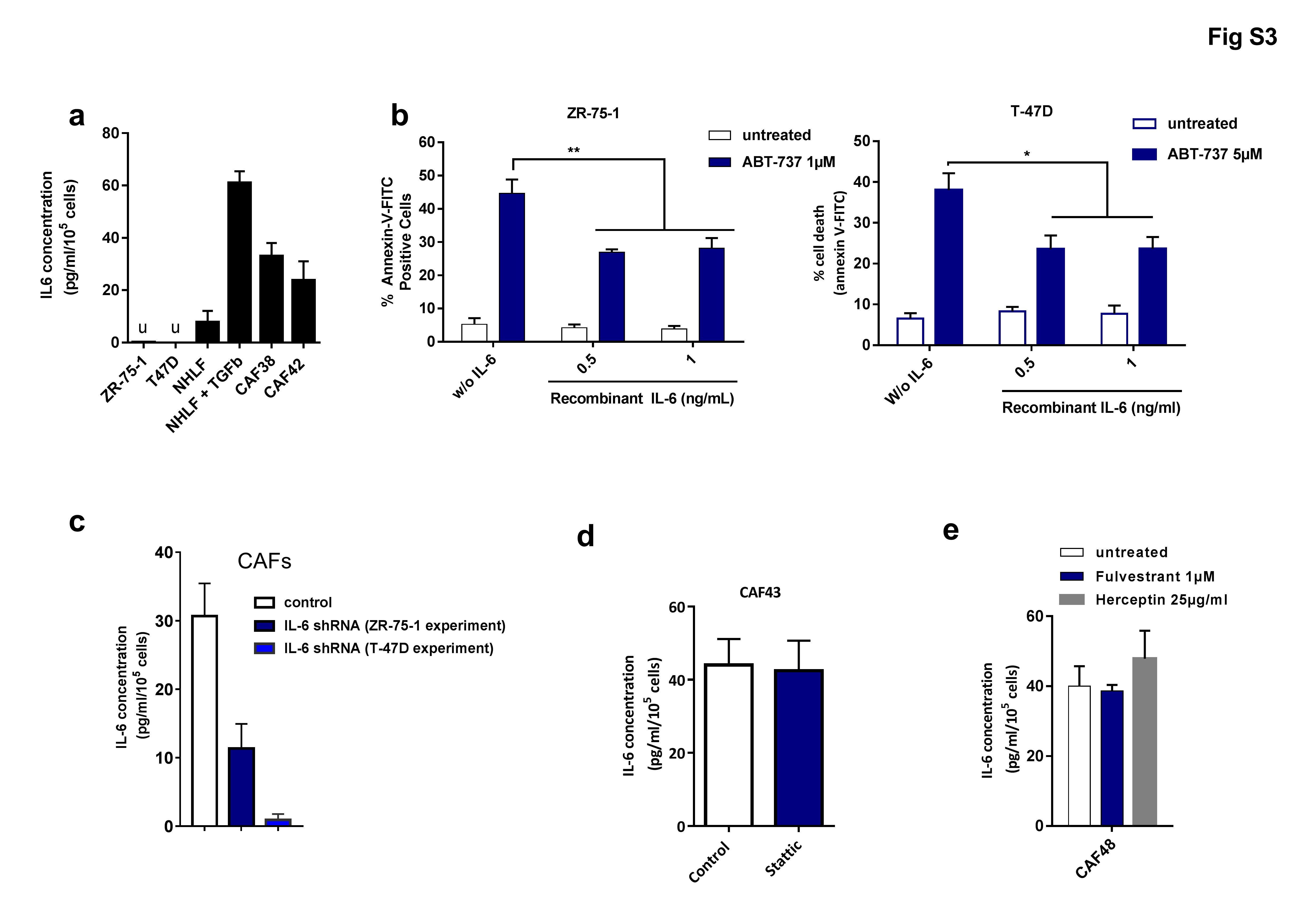

Supplement: Supplementary file 8 — Supplementary Figure 3 [file 41388_2018_635_MOESM8_ESM.tif]
